# Social and economic inequality in coronavirus disease 2019 vaccination coverage across Illinois counties

**DOI:** 10.1038/s41598-021-97705-6

**Published:** 2021-09-16

**Authors:** Tim F. Liao

**Affiliations:** Department of Sociology, University of Illinois at Urbana-Champaign, Urbana, IL 61801 USA

**Keywords:** Disease prevention, Health care economics, Health policy, Health services, Public health

## Abstract

Prior research has well established the association of ethno-racial and economic inequality with COVID-19 incidence and mortality rates across counties in the US. In this ecological study, a similar association was found between ethno-racial and economic inequality and COVID-19 full vaccination rates across the 102 counties in the American state of Illinois in the early months of vaccination. Among the counties with income inequality below the median, a county’s poverty rate had a negative association with the proportion of population fully vaccinated. However, among the counties with income inequality above the median, a higher percentage of Black or Hispanic population was persistently associated with a lower proportion of fully vaccinated population over the two-month period from early February to early April of 2021.

## Introduction

Today, many countries around the world have actively engaged in COVID-19 vaccination. Is COVID-19 vaccination coverage equally distributed geographically within a political territory? If not, is the distribution of vaccination associated with ethno-racial, economic, or other factors? The association of social and economic inequality, especially in terms of county-level ethno-racial and income inequality, with COVID-19 incidence and mortality in the United States is well established^1^. Recent research suggests that Black and Hispanic minorities were also underrepresented in COVID-19 vaccine clinical trials^2,3^. Furthermore, a community’s structural factors such as income inequality may shape health inequities^4^. However, there has been little serious research on social inequality in COVID-19 vaccination beyond clinical trials.

**Figure 1:**
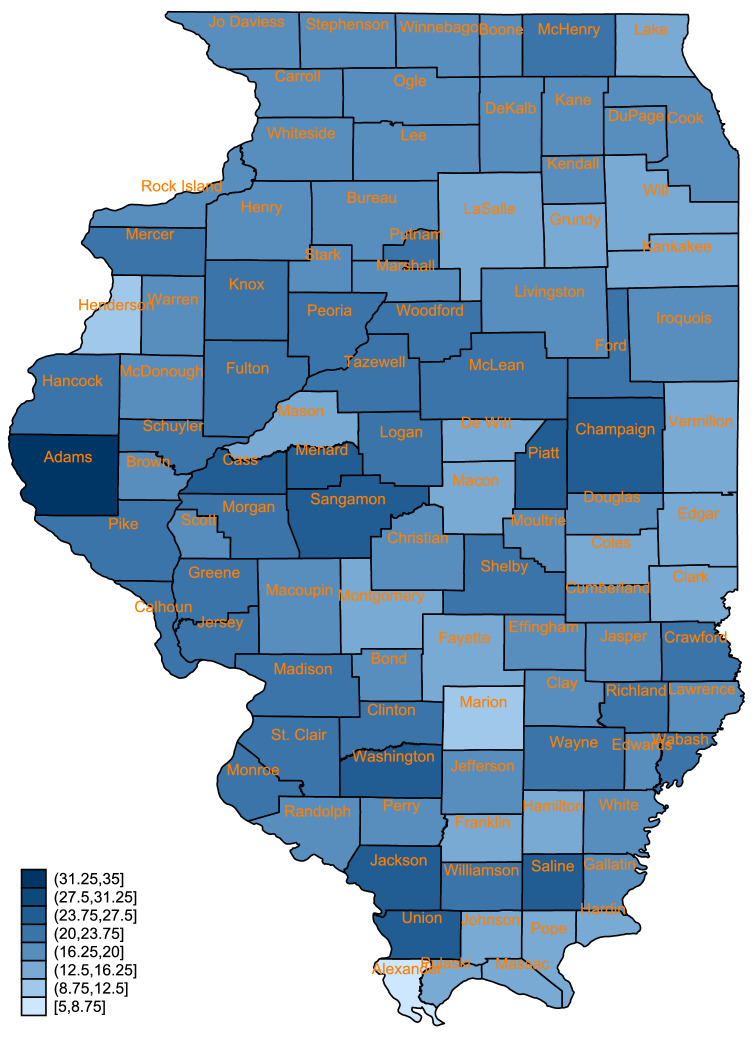
Percent fully vaccinated population across Illinois counties as of April 3, 2021. The map was generated with the command grmap, Stata version 16.0 (stata.com).

The objective of this research is to analyze Illinois county-level association between ethno-racial composition and full vaccination rate conditional on income inequality at three time points between early February and early April of 2021. It is important to analyze full vaccination rates because only when one is fully vaccinated, the risk of COVID-19 infection is drastically reduced. Local mechanisms for providing either one or both dosages of vaccination typically remains the same. The state of Illinois began its Phase 1A vaccination program for frontline healthcare workers on December 15, 2020 and for nursing home and long-term care residents on December 28, 2020. It then started its Phase 1B program for frontline essential workers and residents age 65 and over on January 25, 2021. Its 1B+ program began on February 25, 2021 for additional eligible Illinois residents under age 65 with specific health-complicating conditions. On April 12, 2021, its Phase 2 program was initiated for all residents age 16 and above. Unlike some other American states that did not release systematic vaccination coverage data regularly in the earlier months up to April 2021, Illinois has been releasing county-level COVID-19 vaccination coverage data daily. Figure 1 presents the ecological inequality of percent county population full vaccinated by April 3 (when 18.6% of the population was fully vaccinated in the state), with a range of 25 percentage points between the two counties with the lowest and the highest coverage percentages (7.0% fully vaccinated for Alexander County that has the state-wide highest 31.8% Black population and 31.6% fully vaccinated for Adams County that has a low 3.9% Black and a low 1.8% Hispanic population).

## Methods

The data analyzed are for the 102 counties in the state of Illinois as of February 4, March 10, and April 3, 2021, when 2.6%, 10.8%, and 18.6% of the state’s population were fully vaccinated (with an approximately 8% increment between the first and the second, and the second and third dates). The data sources for the covariates of percent Black, percent Hispanic, Gini inequality index, percent Republican vote, percent male population, and population density are taken from the same sources as in a previous study^1^ and percent population age 65 and over, percent without health insurance. and poverty rate are from two additional sources^5,6^. Table 1 presents a complete list of the data sources. The outcome variable, reported by the Illinois Department of Public Health (IDPH)^7^, is the fraction of a county’s fully vaccinated population. The value of the outcome for Cook County is recalculated. The county has many municipalities including Chicago. Because most other agencies report data for Cook County as a whole while IDPH releases vaccination data separately for Chicago and for the rest of Cook county, the populations fully vaccinated and their total resident populations in the two places were combined for computing a single vaccination fraction for the county.

**Table 1:** The data sources of the 102 Illinois counties.

Variable	Definition	Source
Vaccinated	Fraction of population fully vaccinated	Illinois Department of Public Health
% male	Percent male population, 2019	US Census Bureau
% Age ≥ 65	Percent population age 65 and over, 2019	indexmundi.com
Pop density	Population density per km^2^, 2019	US Census Bureau
% Black	Percent Black population, 2019	US Census Bureau
% Hispanic	Percent Hispanic population, 2019	US Census Bureau
Gini index	Gini index of income inequality	2018 Am. Com. Survey
% Poverty	Percent population in poverty, 2019	Indexmundi.com
% Uninsured	Percent population uninsured, 2020	Countyealthrankings.org
% GOP vote	Percent 2016 GOP vote in a county	GitHub

To model a fractional outcome, a fractional logit model was applied^8^. Fractional regression is appropriate here for analyzing a fractional outcome variable. Fractional logit model is very similar to the standard logit model, with the difference in that the latter requires a dichotomous response of {0, 1} whereas the former is for analyzing a continuous response in the range of [0, 1]. Places with higher income inequality tend to show a heightened association of ethno-racial inequality with COVID-19 outcomes^1^. Therefore, the data were divided into two halves by the Gini inequality median (43.2%). The median is used because it divides the sample into two halves of equal sizes and more importantly because income inequality in the range up to the lower 40% is typically considered little to moderate while above the level, more serious and severe. The 51 counties with lower inequality tend to be less populated, with an average population size of 45.9 thousands whereas the 51 counties with higher inequality tend to be more populated, with an average population size of 202.5 thousands.

The analysis of full vaccination included a set of key factors (percent Blacks, percent Hispanics, income inequality, political affiliation, and poverty) and controls (percent male population, percent population 65 and over, and population density) considered in previous research^1–4,9,10^. An additional control, percent uninsured population, was included in the analysis, for the reason that when health insurance handles the vaccination operation as is the case in some localities, people without insurance may find it difficult to receive vaccination until when it becomes available to the entire resident population. The data were analyzed for each of the income inequality halves separately for the three dates using Stata version 16.0.

## Results

This analysis modeled the association between fully vaccinated population fractions of 102 Illinois counties and percent Black and Hispanic population, percent voting Republican, and poverty rate in a county, together with the four control variables of percent population age 65 and over, percent male population, population density, and percent without health insurance, conditional on income inequality, with the outcome variable measured on three occasions.

**Figure 2:**
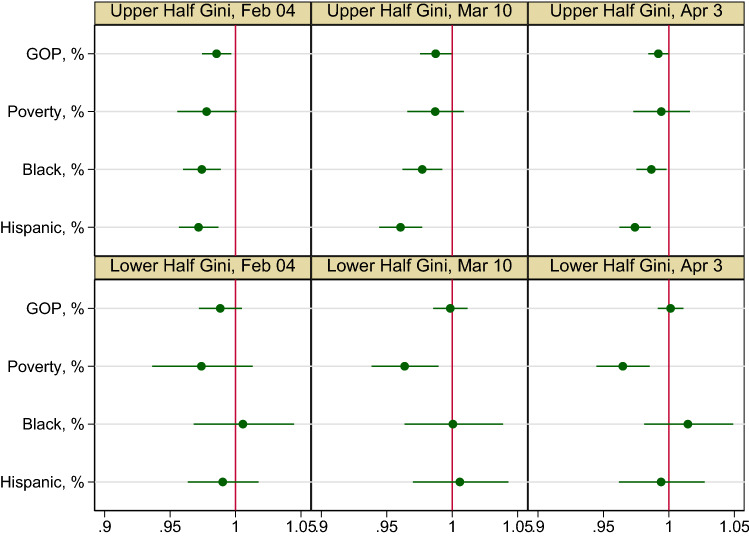
Odds ratios for key covariates in the fractional logit model of fully vaccinated fractions among Illinois counties as of February 4, March 10, and April 3, 2021. The odds ratios for the control variables of population density, percent population age 65 and over, percent male population, and percent uninsured population are not shown; GOP = Republican.

Figure 2 presents odds ratios (OR) for the four key covariates by Gini inequality half. With the other four variables under control, when a county has lower income inequality (represented by the lower panel of the figure), a 1% increase in its poverty rate corresponds to a 3.6% (95% CI 1.0% and 6.2%) decrease in the county population’s odds of being fully vaccinated by March 10 and a 3.5% (95% CI 1.5% and 5.5%) decrease by April 3 although with no significant association by February 4. When a county has higher income inequality (represented by the upper panel of the figure), a 1% increase in a county’s Black population corresponds to a 2.6% (95% CI 1.1% and 4.0%) decrease in the county population’s odds of being fully vaccinated by February 4, a 2.3% (95% CI 1.0% and 3.8%) decrease by March 10, and a 1.3% (95% CI 0.2% and 2.8%) decrease by April 3. Similarly, when a county has higher income inequality, a 1% increase in a county’s Hispanic population corresponds to a 2.8% (95% CI 1.3% and 4.3%), a 3.9% (95% CI 2.3% and 5.6%), and a 2.6% (95% CI 1.4% and 3.8%) decrease in the county population’s odds of being fully vaccinated by the three dates, respectively. When income inequality is high, Republican inclination also displays a moderately negative association with vaccination on the three dates, a 1% increase in a county’s Republican leaning public corresponding to a 1.4% (95% CI 0.3% and 2.6%), a 2.5% (95% CI 0.1% and 2.5%), and a 0.8% (95% CI 0.04% and 1.6%) decrease in the county population’s odds of being fully vaccinated by the three dates, respectively.

**Figure 3:**
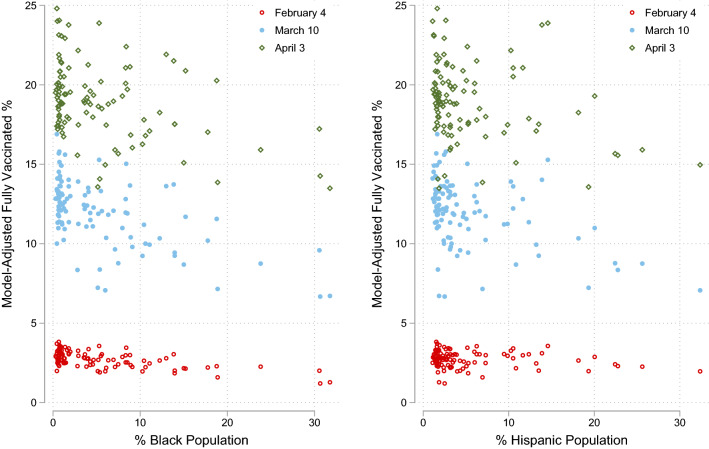
Model-adjusted fully vaccinated rates among the 51 Illinois counties with higher income inequality as of February 4, March 10, and April 3, 2021.

Whereas the estimated associations in terms of ORs and their CIs suggest the size of such associations and related heterogeneities, to obtain a better sense of the statistical properties especially for those counties in the higher half of inequality distributions, the model-adjusted fully vaccinated percentages for the 51 counties are presented in Fig. 3. We can make three observations from the figure. First, regardless of the ethno-racial group or the date, there exists a negative association between the percentage of a county’s minority population and its full vaccination rate. Second, this association appears to be a little tighter for percent Black population than for percent Hispanic population. Third, there appears to be a greater amount of heterogeneity among the 51 counties when the overall vaccination rate is higher at a later time than when it is lower at an earlier time, especially for those counties with a lower ethno-racial composition. A note about a couple of counties with the highest minority populations is in order. In the left panel of the figure, the aforementioned Alexander County (with 31.77% Black population, the highest of the state) shows a near lowest model-adjusted vaccination rate on the first two dates and the lowest rate on the final date. In the right panel, the outlier county across all three dates is Kane County (with 32.37% Hispanic population). However, although it is not the county with the lowest model-adjusted rate across the three dates, it is among the group with the lowest rates. This observation cannot be obtained from Fig. 2 of the estimated ORs presented earlier.

## Discussion

Prior research identified the association of ethno-racial and economic inequality with COVID-19 incidence, mortality, and its vaccine trial inclusion in the United States^1–3^. The current research found similar association between ethno-racial composition and the likelihood of full vaccination in Illinois where economic inequality is high. When a place reaches full vaccination of its entire population, vaccination inequality should disappear. The association between a county’s Black population percentage and vaccination rate reduced a little over time but was still strongly negative by the third date. The same association for the Hispanic composition remained sizable throughout the two-month period. Income inequality suggests unequal access to vaccination, at least in part due to internet vaccination registration in most communities. Public health officials should consider broaden the means of vaccination registration and provide mobile vaccination service to poorer neighborhoods.

The association between partisanship and vaccination suggests a different type of mechanism. Past research found that Democrats were more willing to get vaccinated than Republicans^9,10^. The analysis here confirmed such an association for all three dates in counties with greater income inequality. To obtain a higher rate of participation in COVID-19 vaccination, public health officials may consider highlighting the scientific values, safety, and effectiveness of vaccines for shortening the duration of the pandemic before an eventual return to a normal, unconfined life.

The analysis of the 51 counties with higher income inequality presents a puzzling pattern: In the early stages of vaccination in Illinois, the key factors of percent minorities, poverty, and GOP vote appear to behave differently. The GOP and poverty association with vaccination in terms of odds gravitate toward unity over time whereas percent ethno-racial populations tend to be more persistent. Although the true mechanisms may not be easily discovered, one may speculate that both percent Blacks and percent Hispanics are related to the structural setup of a county and its provision of health services, and that such structural barriers are difficult to remove over a short time. Such barriers may not be so obvious especially during the early stages of a vaccination campaign when a state’s overall full vaccination rate was likely driven by those counties with a smaller ethno-racial population without such barriers. In contrast, whether one voted for the GOP before and whether one was in poverty represents individual factors such as one’s unwillingness or inability to obtain vaccination, and when a county reaches a higher level of vaccination, the influence of such individual factors may gradually disappear. A proviso is that if a large number of individuals are resistant to receiving vaccination in a county due to political reasons, that county may never have its entire population vaccinated.

Why would the factors associated with vaccination rates differ between those counties with higher income inequality versus those with lower inequality levels? As presented in the results section, percent minority populations are associated with vaccination rates when income inequality is higher. There are two possible explanations for the association: First, counties with a higher concentration of minority populations tend to have higher economic inequality, and this is particularly true for percent Black population, with the mean percent Blacks value of those counties with higher income inequality three times greater than the counties with lower income inequality. Second, income inequality tends to have a compounding effect on ethno-racial inequality, as noted in prior research^1^. For those counties with lower income inequality, minority population proportions are typically low. Consequently, factors such as poverty may emerge to the fore as a barrier to vaccination because counties with a high level of poverty may lack resources to speedily initiate vaccination programs.

As noted in the results section, the counties with the highest minority populations, Alexander County and Kane County in the group with higher income inequality, have consistently had low vaccination rates across the three dates. Alexander County is the gateway to the South in the state with a high concentration of Blacks while Kane County is west of Chicago, with a high proportion of Hispanic population. Kane County can be an interesting case in point. Whereas it has one of the lower model-adjusted vaccination rates across the three times, a few counties with higher Black populations show even lower rates than Kane County. This suggests that counties with a higher Black population may face a greater structural barrier than those with a higher Hispanic population.

The research has two relative limitations. First, the data are limited to just one American state, due to the different practices of releasing daily COVID-vaccination information to the public during the early months of vaccination. Although it would be desirable to follow the vaccination coverage in a state like Illinois for a longer time to obtain a dynamic view of vaccination inequalities, the current paper provides an informative analysis of the early, crucial phase of a state’s vaccination effort. Additional, follow-up analysis will be able to reveal the potential saturation point of the ethno-racial, economic, and partisan association found in this research for a greater number of regions when such data become available.

## Data Availability

The corresponding author had full access to all the data in the study and takes responsibility for the integrity of the data and the accuracy of the data analysis. Interested readers can write to the corresponding author to request a copy of the data.
